# Evaluation of Silica-Coated Insect Proof Nets for the Control of *Aphis fabae, Sitophilus oryzae,* and *Tribolium confusum*

**DOI:** 10.3390/nano10091658

**Published:** 2020-08-24

**Authors:** Paraskevi Agrafioti, Sofia Faliagka, Evagelia Lampiri, Merle Orth, Mark Pätzel, Nikolaos Katsoulas, Christos G. Athanassiou

**Affiliations:** 1 Laboratory of Entomology and Agricultural Zoology, Department of Agriculture, Crop Production and Rural Environment, University of Thessaly, Phytokou str., 38446 Volos, Magnesia, Greece; elampiri@agr.uth.gr (E.L.); athanassiou@agr.uth.gr (C.G.A.); 2 Laboratory of Agricultural Constructions and Environmental Control, Department of Agriculture Crop Production and Rural Environment, University of Thessaly, Phytokou Street, 38446 Volos Magnesia, Greece; sofia.faliagka@gmail.com (S.F.); nkatsoul@uth.gr (N.K.); 3 Institut für Textiltechnik der RWTH Aachen University, Otto-Blumenthal-St. 1, 52074 Aachen, Germany; merle.orth1@gmail.com (M.O.); mark.paetzel@ita.rwth-aachen.de (M.P.)

**Keywords:** net formulations, stored product insects, knockdown, mortality, long-term effect

## Abstract

Insect proof nets are widely used in agriculture as mechanical and physical barriers to regulate pest populations in a greenhouse. However, their integration in the greenhouse ventilation openings is highly associated with the decrease of air flow and the adequate ventilation. Thus, there is need for alternative pest management tools that do not impair adequate ventilation. In the present study, we tested four net formulations of relatively large mesh size coated with SiO_2_ nanoparticles, namely, ED3, ED3-P, ED5, and ED5-P to evaluate their insecticidal properties against adults of *Aphis fabae* and *Sitophilus oryzae* and larvae of *Tribolium confusum*. ED3 and ED5 nets were coated with SiO_2_ nanoparticles of different diameter, while in the case of ED3-P and ED5-P, paraffin was added to increase the mass of the deposited particles on the net’s surface. In the first series of bioassays, the knockdown and mortality rates of these species were evaluated after exposure to the aforementioned net formulations for 5, 10, 15, 20, 25, 30, 60, 90, and 180 min. In the second series of bioassays, knockdown and mortality of these species were recorded after 1, 7, and 10 days of post-exposure to the nets for different time intervals (15, 30, and 60 min). Based on our results, all nets significantly affected *A. fabae,* since all insects were dead at the 1-day post-exposure period to the silica-treated nets. Conversely, at the same interval, no effect on either *S. oryzae* adults or *T. confusum* larvae was observed. However, in the case of *S. oryzae*, the efficacy of all nets reached 100% 7 days after the exposure, even for adults that had been initially exposed for 15 min to the treated nets. Among the species tested, *T. confusum* larvae exhibited the lowest mortality rate, which did not exceed 34% at the 10 days of post-exposure interval. Our work underlines the efficacy of treated nets in pest management programs, under different application scenarios, at the pre- and post-harvest stages of agricultural commodities.

## 1. Introduction

Despite the association of many insecticidal compounds with mammalian toxicity and increased environmental footprint, conventional pesticides are widely used as a means of pest management. Over the years, many insects species have developed resistance mechanisms to the vast majority of active ingredients that are used as synthetic insecticides [1,2,3]. According to the European Commission, a sustainable and environmentally friendly insecticidal strategy should be followed in the case of adequate crop protection, to minimize the negative effects of the intensive chemical pesticide applications on both natural enemies (predators, parasitoids) and human health [4,5]. Thus, the notion of integrated pest management (IPM) has now become entrenched in agriculture.

Nets are widely used in agriculture as mechanical or physical barriers in order to protect crops from either biotic or abiotic stresses. In the Mediterranean region, protection against insects is considered significantly important even compared to the management of excessive heat during the summer [6]. Insect proof nets have been integrated in greenhouse ventilation openings as an alternative to pesticides for many years and their use is related to the management of the population density of external agents. At the same time, exclusion nets are used to cover entire greenhouse structures or screenhouses [7]. Nets have a dual effect as they block the penetration of pests, while prevent the escape of insects used for crop pollination [8]. The design of nettings is determined by the size and geometry of the hole, the size of the insect thorax, as well as the method of knitting [9].

According to Kittas et al. [6], the environment of a greenhouse is qualified by significant heterogeneity in which various factors contribute, such as the crop itself, the cover materials and the insect screens. Microclimate conditions of a greenhouse are strongly affected by nets’ physical and optical properties [10]. Very fine mesh size nets severely affect the ventilation and air capacity of the enclosed area by decreasing permeability [11,12]. Indeed, the ventilation rate can be decreased by 40–50% when anti-aphid or anti-thrip nettings are applied [11,13]. These results are in accordance with those of Katsoulas et al. [14], who indicated a 33% decrease of ventilation flow due to the installation of an anti-aphid insect screen with 50% porosity on the vents of a polyethylene-covered greenhouse. In addition, Baeza et al. [15] showed that the use of an anti-insect screen with porosity of 25%, which is commonly used to exclude white flies *Bemisia tabaci* (Gennadius) (Hemiptera: Aleyrodoidae) and the western flower thrips *Frankliniella occidentalis* (Pergande) (Thysanoptera: Thripidae), could lead to a ventilation reduction of approximately 88%. As a result, higher temperature regimes are recorded in the interior of the protected greenhouse plants leading to non-uniform production, increased yield losses, as well as quality deterioration [6,14]. Small mesh size nets (i.e., low porosity nets) may contribute to undesired results, since they exclude not only harmful pests but also beneficial insects [16]. Moreover, Bell and Baker [11], showed that the migration of insects in a greenhouse is not always correlated to the mesh size, since insects with very small thoracic width, such as whiteflies, can intrude the finest pore size net.

The establishment of insect proof nets for the stored product pest management is directly linked to environmental protection, as their use does not lead to residues or to contamination in storage facilities and stored commodities [17]. Many studies are focused on the design of long-lasting insecticide- treated nets (LLINs) by incorporating insecticides into the net coating in order to minimize the chemical sprays directly on the crop [18,19]. Dáder et al. [18] showed that LLINs caused a significant reduction in *Myzus persicae* (Sulzer) (Hemipetra: Aphidedae) and *Aphis gossypii* (Glover) (Hemipetra: Aphididae) immigration without affecting the parasitoid *Aphidius colemani* Viereck (Hymenoptrea: Aphididae). However, during field trials, LLINs effectiveness was degraded after sun exposure whereas, the efficacy of either bifenthrin or deltamethrin was suggested as insufficient to control *B. tabaci* culture due to its small size. Notwithstanding, according to Arthurs et al. [19], nettings of large mesh size treated with deltamethrin proved insufficient to protect a greenhouse crop from *F. occidentalis* invasion. Hence, both the effect of LLINs on beneficial enemies and their effectiveness in small sized insects should be further evaluated [18].

Recently, Rumbos et al. [17] successfully demonstrated the efficacy of Carifend^®^, a polyester net coated with alpha-cypermethrin, against two stored tobacco insect pests, the cigarette beetle, *Lasioderma serricorne* (F.) (Coleoptera: Anobiidae) and the tobacco moth, *Ephestia elutella* (Hübner) (Lepidoptera: Pyralidae). Moreover, Paloukas et al. [20] suggested that Carifend^®^ could be effectively used to control different major stored-product insects such as the rice weevil, *Sitophilus oryzae* (L.) (Coleoptera: Curculionidae)*,* since 98% mortality was recorded two weeks after the exposure to the treated net.

The use of nanoparticles as a novel biological insect control agent has been well established in the recent years. Silicon is known as the most frequent metalloid and the most copious element on earth after oxygen [21,22,23]. The mode of action of silicon dioxide (SiO_2_) begins with the adherence of the nanoparticles on the cuticle of the insect, inactivating epicuticular lipids, which leads to death through desiccation [24,25]. Furthermore, Rastogi et al. [22], suggested that the mortality of pests could be related to congestion of spiracles and tracheas caused by the nanosilica particles.

Diatomaceous earth (DE) that consists mainly of amorphous hydrated silica has been extensively used in stored product insect management. Vayias and Athanassiou [26] stated that the effectiveness of DE is associated with the developmental stage of the insects. Indeed, the management of the confused flour beetle adults, *Tribolium confusum* Jacquelin DuVal (Coleoptera: Tenebrionidae) is considerably difficult compared to soft-bodied greenhouse pests such as aphids. On the other hand, aphids are among the most common pests in horticultural crops and are related to the transmission of many economically hazardous viruses even after injecting their sucking mouthparts on the leaves for a very short time interval [18,27]. Unlike hard-bodied beetles, aphids are considered to be much more vulnerable to desiccation from sorptive materials and micro-wounds. For instance, Singh and Singh [28] found that DEs could cause rapid mortality of the aphid *Rhopalosiphum padi* (L.) (Homoptera: Aphididae), when applied on wheat plants. However, in that study, the authors stood eventually against these DE applications, because of negative photosynthetic rates [28].

Recently, Shoaib et al. [29], indicated that the exposure of larvae of *Plutella xylostella* (L.) (Lepidoptera: Plutellidae) to 1 mg × cm^−2^ of a siliceous dust formulation resulted in 85% mortality after 72 h. The authors suggested that the mortality rate is improved as dose and exposure interval are increased. Moreover, silica nanoparticles, concerning their physical and chemical properties, differ significantly from their bulk formation [19]. According to Debnath et al. [30], nanoparticles proved to be much more effective when compared with bulk silica against *S. oryzae*. At the post-harvest stages of agricultural commodities, stored product protection could be enhanced by the use of silicon-based nanoparticles as non-conventional pesticide agents [2,31].

To date, little attention has been given to the incorporation of physical inert dusts to the yarns of insect proof nets. The application of non-toxic and environmental-friendly nets could replace the insecticidal treated nets leading to insecticide-free products. Thus, the objective of our study was to assess the effectiveness of silicon dioxide-treated nets under laboratory conditions for the control of the black bean aphid, *Aphis fabae* Scopoli (Hemipetra: Aphididae), and the stored product pests *T. confusum* and *S. oryzae.* The aforementioned species were exposed to insect proof nets coated with two different silicon dioxide dust formulations, Syloid^®^ED3 and Syloid^®^ED5 with and without the addition of paraffin in order to achieve better particle adherence to the net, respectively.

## 2. Materials and Methods

### 2.1. Insects Tested

The rearing of the *A. fabae* individuals took place at the Laboratory of Agricultural Entomology, Benaki Phytopathological Institute, Attica, Greece. Aphids were reared on *Vicia faba* L. plants at 20 ± 1 °C, relative humidity (r.h.) of 65 ± 2% and a photoperiod of 16 h in light: 8 h in dark [32]. Aphid colonies were transferred to the Laboratory of Entomology and Agricultural Zoology (LEAZ), Department of Agriculture, Crop Production and Rural Environment, University of Thessaly to carry out the bioassays.

Established laboratory colonies of *S. oryzae* and *T. confusum* were maintained in LEAZ under controlled conditions in a growth chamber as suggested by Faliagka et al. [33].

### 2.2. Nettings and Dust Formulations

Experiments were carried out with four samples of the same insect proof net, the textiles of which were performed by the Institut für Textiltechnik (ITA) of RWTH Aachen University. The weaving and the evaluation of the nets was provided from Thrace Nonwovens & Geosynthetics S.A. (Thrace, Greece). All textiles were coated either with Syloid^®^ ED3 or Syloid^®^ ED5 which are amorphous silica dust formulations (P&S Powder and Surface GmbH, Salzkotten, Germany). The former silica dust consists of 99–100% SiO_2_ with particle size of 5.8 μm, while the latter consists of 95–100% SiO_2_ with particle size of 9.0 μm. Paraffin was added as an organic primer in two of the four netting samples (ED3-P and ED5-P) consisting of different silica formulation in order to enhance the adhesion properties of each dust to the net. ED3 and ED5 samples were used without the addition of paraffin. The purpose of the primer is the integration of up to 40% more SiO_2_ particles on the net. The mass of the deposited silica particles on the net’s surface was fluctuated from 0.4 to 0.9 g × m^−2^ depending on the dust formulation as well as the addition of paraffin. However, paraffin may reduce the mesh size and therefore, the air permeability properties of the tested nets. In the present study, the insecticidal properties of SiO_2_ nanoparticles were evaluated. In the case where adequate results are provided, it is suggested to incorporate silica on nets of larger pores in order to alleviate insufficient ventilation. Table 1 shows the properties of the nets used in the experimental trials.

All samples were produced by the same 50 mesh net that was constructed by using high density polyethylene (HDPE) monofilament yarns. SiO_2_ free-nettings with the same mesh size as well as clear Petri dishes without the addition of nets were used as a control treatment. The silica coating procedure was repeated two times for each tested net.

### 2.3. Bioassay Series

Nets were fine cut and adjusted at the bottom of plastic Petri dishes (59.4 cm^2^ in surface), using a thin layer of silicon. The lids of plastic dishes are usually loose-fitting making it easy for insects to escape, so, the “neck” of each Petri dish was covered with polytetrafluoroethylene dispersion, (Northern Product, Cumberland, RI, USA). A single fine brush was used to insert the species into the Petri dishes to avoid wounding.

#### 2.3.1. Short-Term Effect

In the short-term effect bioassay series, adults of *A. fabae* and *S. oryzae*, and larvae of *T. confusum* were used. Ten insects of each species were transferred to the Petri dishes. All species were exposed to silica-treated nets, clear Petri dishes (without nets), and untreated nets for the intervals of 5, 10, 15, 20, 25, 30, 60, 90, and 180 min. For each exposure interval, mortality and knockdown effect of all species in each Petri dish were recorded. For each of the four nets, the bioassay was replicated eight times per exposure interval. Thus, in this series of bioassays 144 Petri dishes were used in total.

#### 2.3.2. Long-Term Effect

The purpose of this series of bioassays was to evaluate the effect of the nets on the knockdown and mortality rates of the exposed individuals at certain post-exposure intervals. Thus, as above, ten individuals of each species were transferred to Petri dishes, with different plates per species, and exposed to the nets for 15, 30, and 60 min. Knockdown and mortality were recorded immediately after the completion of the exposure periods and then all insects were carefully transferred to new dishes consisting of untreated nets or clear Petri dishes (without nets). When transferring the insects, a clean single fine brush was used to avoid possible contamination of the dust in the treated nets. Within each exposure interval of 15, 30, and 60 min no food was added in the Petri dishes. However, during post-exposure period for *S. oryzae* and *T. confusum,* cracked wheat (0.5 ± 0.1 gr) and wheat flour (1.0 ± 0.1 gr) were added in all petri dishes, respectively. In the case of *A. fabae* no food was supplied, since the bioassay lasted one day, unlike that of the stored product insects which was completed after 10 days. All bioassays were carried out at 25 °C and 65% r.h. Mortality and knockdown effect of *S. oryzae* and *T. confusum* were assessed after 1, 7, and 10 days, whereas in the case of *A. fabae* after 1 day. For each of the four nets, the bioassays were replicated eight times. A total of 624 Petri dishes were used to perform this series of bioassays.

### 2.4. Statistical Analysis

The results of the first series of bioassay (short-term effect) were analyzed using Probit Analysis to estimate the knockdown time, i.e., KD_t50_, KD_t95_, and KD_t99_ for each species and net formulation. The intervals tested yielded a variety of knockdown results only for *A. fabae*, which allowed the calculation of KD_t50_, KD_t95_, and KD_t99_ values. For the second series of bioassays, separately of each species, the data (knockdown and mortality) were converted to percentages and then were submitted to a two-way ANOVA, to determine the differences among the exposure intervals (15, 30, and 60 min) and among the net formulations (including controls as well), while means of knockdown and mortality were separated using the Tukey-Kramer HSD test at the 5% level. Moreover, the same approach was conducted for the long-term effect for each species tested. The data were analyzed using SPSS 25.0 (SPSS Inc., Chicago, IL, USA).

## 3. Results

### 3.1. Short-Term Effect

The mortality rate of all tested species was relatively low. In the first series of tests, KD_t_ values were not found to fit the data well, since *P* values were <0.01 (Table 2). In most of the cases, confidence interval could not be estimated for *A. fabae* and *S. oryzae*, except for ED5-P of *A. fabae*. For *T. confusum*, Probit could not be estimated, since the percentage of knocked down individuals was negligible (data not presented).

Overall, KD_t99_ values for *A. fabae* at ED3-P resulted in the highest values among treatments, corresponding to 1590.9 min (approximately 26 h) (Table 2). Contrariwise, the lowest KD_t99_ value was recorded at ED5-P, corresponding to 363.3 min (approximately 6 h) (Table 2). Moreover, for *S. oryzae* the resulting values were similar with those of *A. fabae* at ED3, which were 1235.7 min (approximately 20 h) and 1281.4 min (approximately 21 h), respectively. For KD_t50_ the lowest value (51.3 min) for *A. fabae* was recorded at ED5, whereas the highest value (279.7 min) was noted at ED3-P (Table 2).

### 3.2. Long-Term Effect

The mortality rate ranged significantly between the tested cases (Table 3, Table 4 and Table 5). Knockdown of *A. fabae* was low and did not exceed 28.7% (Table 3), whereas mortality was negligible (data not presented). For knocked down individuals, significant differences were noted in all tested net formulations among the time intervals (15, 30, and 60 min). Finally, at the 1-day post-exposure period, mortality was 100% in all tested net formulations (Table 3).

Regarding *S. oryzae* individuals, knockdown and mortality were negligible, in all tested net formulations for the 15, 30, and 60 min intervals (data not presented), while at the 1-day post-exposure period, no significant differences were noted among the exposure intervals (Table 4). At this interval, mortality was generally low, reaching 6.2%. However, in most of the cases, at the 7-day post-exposure period mortality was approximately 87%, even when the initial exposure was 15 min, while after 10 days mortality reached 100% (Table 4).

Regarding *T. confusum* larvae, knockdown and mortality were generally low (<5%, data not presented). Larval knockdown of *T. confusum* was also negligible (data not presented). After 7 d of exposure in all formulations, mortality did not exceed 30%, whereas after 10 d the highest mortality level was 34% at ED5-P (Table 5). Significant differences were recorded among the exposure intervals in most of the cases (Table 5).

## 4. Discussion

The results of our study indicated that even in the case of the longest exposure interval (180 min) no immediate mortality was achieved for either of the stored product species tested. However, for *A. fabae*, knockdown effect was vigorous and rapid, especially at longer intervals, as KD_t50_ was high after approximately one hour. In general, from the most to the least susceptible, the species/life stages tested here can be classified as *A. fabae* adults > *S. oryzae* adults > *T. confusum* larvae.

Particle size is highly associated with the insecticidal efficacy of inert dusts. Many authors have emphasized on the importance of the particle size, a significant physical property of dust formulations, underlining that formulations of smaller particles may enhance the insecticidal efficacy on a variety of stored-product beetles [34,35,36,37,38,39]. Korunić [40] found that the efficacy of DEs is promoted when particle size ranges between 1 and 30 μm. Similarly, Vayias et al. [38] indicated that DEs particles, sized up to 45 μm, resulted in higher mortality of stored product beetles, as compared with larger particles. However, Korunić [40] underlined the significance of the DEs origin, and concluded that even a high range between the size of particles (i.e., 0–192 μm) could lead to similar insecticidal results, indicating the significance of the particle shape. In this context, Rumbos et al. [39] found that the insecticidal effect of zeolites on the control of stored-product pests was not highly associated with particle size.

Peng et al. [41] stated that the dispersity and stability of SiO_2_ nanoparticles are enhanced when added to liquid paraffin. In the present study, it was concluded that the susceptibility of *A. fabae* and *S. oryzae* was high, regardless of the addition of the primer or the size of silica nanoparticles, since no significant differences were recorded concerning the long-term effect. Notwithstanding, our results suggested that the use of paraffin on the net’s surface contributes to the reduction of fabrics’ shrinkage whereas it increases the rate of the particle deposition. Thus, the knockdown effect of *A. fabae* was increased when insects were exposed to ED5-P nets since in this case, the deposition of silica particles was the highest among all tested samples due to the addition of paraffin. In particular, it was shown that 99% of the adults were knocked down after six hours when exposed to the net coated with the ED5-P dust formulation, which consisted of the largest silica particles at the highest deposition rate.

Silica particles should not be considered as the only lethal factor in our study, as mortality can be also attributed to the presence of the net itself. As shown in the results corresponding to the control treatments, insects exposed to the SiO_2_-free nets were also affected by the presence of the net as opposed to the insects exposed to the dishes without net. Concerning the above, silica-treated nets could be applied on the vent openings of a greenhouse limiting the number of infestations due to greenhouse pests’ invasions, which occurs mechanically, i.e., without the use of the dust. A similar approach has been investigated in the case of stored product insects, with similar results regarding the detrimental effect of the net to the insect bodies [17,20].

Although no immediate mortality was recorded for all the insect species tested, delayed mortality was high, reaching 100% even in the shortest exposure time (15 min). Similarly, Kavallieratos et al. [42] observed a significant delayed mortality on 14 d for *S. oryzae* and the lesser grain borer, *Rhyzopertha dominica* (F.) (Coleoptrea: Bostrychidae) when exposed to a mixture of DE and abamectin. Our data show that, despite low initial effect, delayed mortality for the two beetle species was high, which clearly suggests that the exposed individuals were heavily affected by the presence of the dusts in their cuticles. As inert materials are slow-acting, it has been observed that mortality may take several days or even weeks to occur, even if the insects are in continuous contact with the dust particles [43,44]. Nevertheless, the current results suggest that even the shortest exposure to the dust particles tested here is irreversibly lethal, and recovery is less likely to appear. This is particularly important, as insects will eventually die after their removal from the treated substrate, a characteristic that is even more important in the case of aphids, if it is combined with the knockdown that appears rapidly after exposure.

To conclude, this study indicates that all of the nets tested here were effective for the control of aphids and less for beetles (including larval stage), despite variations in efficacy among treatments. Practically, the nets that are coated with inert materials can serve as a good alternatives to traditional pesticides in greenhouses but also in food and processing facilities, significantly moderating insect immigration. This technology can be adopted in a wide range of facilities, and also in food packaging, where the larval stage can penetrate with ease. At the same time, in a “real world” greenhouse scenario, immigration of aphids from the outside toward the plantation will force the insects to pass through the treated net, which is expected to be lethal, even if some aphids eventually pass successfully.

## Figures and Tables

**Table 1:** Properties of the silicon coated nets ED3 (without paraffin), ED3-P (paraffin), ED5 (without paraffin), and ED5-P (paraffin).

Sample	Mesh Size	Silica Particles Diameter (μm)	Coating Repetition	Mass of Deposited Silica Particles on the Surface of the Net (g × m^−2^)
ED3	50 mesh	5.8	2	0.4
ED3-P	50 mesh	5.8	2	0.7
ED5	50 mesh	9.0	2	0.7
ED5-P	50 mesh	9.0	2	0.9

**Table 2:** Probit analysis for KD_t50_, KD_t95_, and KD_T99_ (confidence intervals) of *Aphis fabae* and *Sitophilus oryzae* adults exposed to four different nets (ED3, ED3-P, ED5, ED5-P) for 180 min, values are expressed as minutes to knockdown.

#	Nets	KD_t50_	KD_t95_	KD_t99_	Slope ± SE	X^2^	*P*
*A. fabae*	ED3	253.3 ^a^	980.2 ^a^	1281.4 ^a^	2.7 ± 0.1	389.8	<0.01
	ED3-P	279.7 ^a^	1206.8 ^a^	1590.9 ^a^	2.1 ± 0.1	290.9	<0.01
	ED5	51.3 ^a^	669.6 ^a^	925.8 ^a^	3.3 ± 0.1	561.7	<0.01
	ED5-P	74.9 (49.8—109.1)	278.8 (206.6—465.8)	363.3 (265.6—619.5)	9.6 ± 0.1	372.8	<0.01
#	**Nets**	**KD_t50_**	**KD_t95_**	**KD_t99_**	**Slope ± SE**	**X^2^**	***P***
*S. oryzae*	ED3	-	-	-	-	-	-
	ED3-P	767.3 ^a^	1098.5 ^a^	1235.7 ^a^	0.6 ± 0.1	449.7	<0.01
	ED5	-	-	-	-	-	-
	ED5-P	-	-	-	-	-	-

^a^ Could not estimate confidence intervals; - Could not estimate knockdown time.

**Table 3:** Short- and long-term knockdown (KD) and mortality (% ± SE) of *Aphis fabae* adults exposed for 15, 30, and 60 min to dishes with four different treated nets (ED3, ED3-P, ED5, ED5-P), dishes with untreated net and dishes without net. The first day after exposure for each exposure interval is considered as the long-term effect.

	ED3			ED3-P			ED5			ED5-P			Untreated net			Without net		
	Short term	Long term		Short term	Long term		Short term	Long term		Short term	Long term		Short term	Long term		Short term	Long term	
Exposure time	KD	KD	Mortality	KD	KD	Mortality	KD	KD	Mortality	KD	KD	Mortality	KD	KD	Mortality	KD	KD	Mortality
15 min	0.0 ± 0.0 a	0.0± 0.0 A	100.0 ± 0.0 A	0.0 ± 0.0 a	0.0 ± 0.0 A	100.0 ± 0.0 A	0.0 ± 0.0 a	0.0 ± 0.0 A	100.0 ± 0.0 A	0.0 ± 0.0 a	0.0 ± 0.0 A	100.0 ± 0.0 A	0.0 ± 0.0	2.5 ± 2.5 A	100.0 ± 0.0 A	0.0 ± 0.0	20.0 ± 3.2 B	36.2 ± 8.6 B
30 min	0.0 ± 0.0 a	0.0 ± 0.0 A	100.0 ± 0.0 A	0.0 ± 0.0 a	0.0 ± 0.0 A	100.0 ± 0.0 A	0.0 ± 0.0 a	0.0 ± 0.0 A	100.0 ± 0.0 A	0.0 ± 0.0 a	0.0 ± 0.0 A	100.0 ± 0.0 A	0.0 ± 0.0	0.0 ± 0.0 A	100.0 ± 0.0 A	0.0 ± 0.0	22.5 ± 13.1 B	12.5 ± 3.1 B
60 min	27.5 ± 4.5 bA	0.0 ± 0.0 A	100.0 ± 0.0 A	22.5 ± 3.1 bA	0.0 ± 0.0 A	100.0 ± 0.0 A	28.7 ± 3.9 bA	0.0 ± 0.0 A	100.0 ± 0.0 A	15.0 ± 4.6 bA	0.0 ± 0.0 A	100.0 ± 0.0 A	0.0 ± 0.0 B	0.0 ± 0.0 A	93.7 ± 4.1 A	0.0 ± 0.0 B	16.2 ± 4.6 B	23.7 ± 6.7 B
F	36.8	-	-	51.5	-	-	52.1	-	-	10.5	-	-	-	1.0	2.2	-	0.1	3.2
*P*	<0.01	-	-	<0.01	-	-	<0.01	-	-	<0.01	-	-	-	0.38	0.13	-	0.86	0.06

Within each exposure interval, post-exposure interval, and net formulation, means followed by the same lowercase letters (a, b) do not differ significantly according to Tukey-Kramer HSD test at *P* < 0.05. Within each exposure interval, post-exposure interval, and net formulation, mortality, and knockdown means followed by the same uppercase letters (A, B) do not differ significantly across treatments according to Tukey-Kramer HSD test at *P* < 0.05. Where no letter exist, no significant differences were noted. ANOVA parameters for long-term effect and knocked down adults were: at 15 min F = 22.8, *P* < 0.01, 30 min, F = 2.9, *P* = 0.02, 60 min, F = 12.4, *P* < 0.01, whereas for short-term effect and knocked down adults at 60 min, F = 22.9, *P* < 0.01. For delayed mortality at 15 min, F = 54.3, *P* < 0.01, 30 min, F = 779.5, *P* < 0.01, 60 min, F = 88.7, *P* < 0.01, in all cases *df* = 2, 23. In the case where “-“is indicated, F and *P* values were not provided in the Table since no variances among the samples were recorded.

**Table 4:** Delayed mortality (% ± SE) of *Sitophilus oryzae* adults exposed for 15, 30, and 60 min to dishes with four different nets (ED3, ED3-P, ED5, ED5-P), dishes with untreated net and dishes without net. The 1st, 7th and 10th day after exposure for each exposure interval is considered as the long-term effect.

	ED3			ED3-P			ED5			ED5-P			Untreated Net			Without Net		
Exposure time (d)	1st	7th	10th	1st	7th	10th	1st	7th	10th	1st	7th	10th	1st	7th	10th	1st	7th	10th
15 min	2.5 ± 1.6	100.0 ± 0.0 A	100.0 ± 0.0 A	0.0 ± 0.0	97.5 ± 2.5 A	98.7 ± 1.2 A	0.0 ± 0.0	100.0 ± 0.0 aA	100.0 ± 0.0 aA	5.0 ± 1.8	100.0 ± 0.0 bA	100.0 ± 0.0 aA	6.2 ± 4.1	40.0 ± 8.4 B	56.2 ± 9.0 B	0.0 ± 0.0	0.0 ± 0.0 C	11.2 ± 2.2 C
30 min	3.7 ± 1.8	97.5 ± 2.5 A	100.0 ± 0.0 A	5.0 ± 1.8	97.5 ± 1.6 A	100.0 ± 0.0 A	0.0 ± 0.0	96.2 ± 1.8 bA	96.2 ± 1.8 bA	3.7 ± 2.6	97.5 ± 2.5 abA	100.0 ± 0.0 aA	2.5 ± 1.6	22.5 ± 10.6 B	30.0 ± 11.0 B	0.0 ± 0.0	0.0 ± 0.0 C	11.2 ± 2.9 C
60 min	2.5 ± 1.6	98.7 ± 1.2 A	100.0 ± 0.0 A	3.7 ± 1.8	100.0 ± 0.0 A	100.0 ± 0.0 A	0.0 ± 0.0	100.0 ± 0.0 aA	100.0 ± 0.0 aA	0.0 ± 0.0	87.5 ± 5.2 aA	88.7 ± 5.4 bA	2.5 ± 2.5	43.7 ± 9.9 B	56.2 ± 9.8 B	0.0 ± 0.0	0.0 ± 0.0 C	12.5 ± 3.1 C
F	0.8	0.6	-	2.9	0.7	1.0	-	4.2	4.2	1.9	3.9	4.2	0.5	1.3	2.3	-	-	0.1
*P*	0.83	0.55	-	0.07	0.50	0.38	-	0.02	0.02	0.16	0.03	0.02	0.59	0.27	0.12	-	-	0.93

Within each exposure interval, post-exposure interval, and net formulation, means followed by the same lowercase letters (a, b) do not differ significantly according to the Tukey-Kramer HSD test at *P* < 0.05. Within each exposure interval, post-exposure interval, and net formulation, mortality means followed by the same uppercase letters (A, B) do not differ significantly across treatments according to the Tukey-Kramer HSD test at *P* < 0.05. Where no letters exist, no significant differences were noted. ANOVA parameters for 1st day were: at 15 min, F = 1.9, *P* = 0.10, 30 min, F = 1.6, *P* = 0.18, 60 min, F = 1.3, *P* = 0.26, for 7th day were: at 15 min, F = 142.2, *P* < 0.01, 30 min, F = 91.9, *P* < 0.01, 60 min, F = 79.1, *P* < 0.01, whereas for 10th day were: at 15 min, F = 92.2, *P* < 0.01, 30 min, F = 75.4, *P* < 0.01, 60 min, F = 55.6, *P* < 0.01, in all cases *df* = 5, 47. In the case where “-“is indicated, F and *P* values were not provided in the Table since no variances among the samples were recorded.

**Table 5:** Delayed mortality (% ± SE) of *Tribolium confusum* larvae exposed for 15, 30, and 60 min to dishes with four different nets (ED3, ED3-P, ED5, ED5-P), dishes with untreated net and dishes without net. The 1st, 7th, and 10th day after exposure for each exposure interval is considered as the long-term effect.

	ED3			ED3-P			ED5			ED5-P			Untreated net			Without Net		
Exposure time (d)	1st	7th	10th	1st	7th	10th	1st	7th	10th	1st	7th	10th	1st	7th	10th	1st	7th	10th
15 min	3.7 ± 2.6	12.5 ± 3.1 ab	13.7 ± 3.2	1.2 ± 1.2 a	8.7 ± 4.4 a	18.7 ± 3.5	2.5 ± 1.6 ab	13.7 ± 4.1	28.7 ± 2.9 a	2.5 ± 1.6	11.2 ± 4.7 ab	18.7 ± 5.4 ab	6.2 ± 2.6	5.0 ± 1.8	16.2 ± 3.7	0.0 ± 0.0	3.7 ± 2.6	28.7 ± 5.1
30 min	0.0 ± 0.0	7.5 ± 2.5 aA	12.5 ± 3.6	2.5 ± 1.6 ab	18.7 ± 3.5 abB	20.0 ± 3.7	0.0 ± 0.0 a	6.2 ± 2.6 A	8.7 ± 2.2 b	0.0 ± 0.0	8.7 ± 2.2 aAB	11.2 ± 1.2 a	2.5 ± 2.5	7.5 ± 2.5 A	18.7 ± 6.3	0.0 ± 0.0	1.2 ± 1.2 A	21.2 ± 3.5
60 min	6.2 ± 1.8 B	20.0 ± 4.2 bAB	22.5 ± 3.6	7.5 ± 1.6 bB	30.0 ± 5.3 bB	32.5 ± 5.2	7.5 ± 2.5 bB	16.2 ± 3.7 AB	25.0 ± 3.7 a	3.7 ± 1.8 AB	26.5 ± 6.5 bB	33.7 ± 7.0 b	3.7 ± 1.8 AB	6.2 ± 3.2 A	15.0 ± 4.2	0.0 ± 0.0A	2.5 ± 1.6 A	22.5 ± 3.1
F	2.9	3.5	2.4	4.7	5.6	3.2	4.9	2.1	12.0	1.8	3.8	4.8	0.7	0.2	0.1	-	0.4	1.0
*P*	0.07	0.04	0.11	0.02	0.01	0.06	0.01	0.14	0.01	0.18	0.03	0.01	0.52	0.79	0.86	-	0.66	0.38

Within each exposure interval, post-exposure interval, and net formulation mortality, means followed by the same lowercase letter do not differ significantly according to the Tukey-Kramer HSD test at *P* < 0.05. Within each exposure interval, post-exposure interval, and net formulation, mortality means followed by the same uppercase letter do not differ significantly across treatments according to the Tukey-Kramer HSD test at *P* < 0.05. Where no letters exist, no significant differences were noted. ANOVA parameters for 1st day were: at 15 min, F = 1.3, *P* = 0.6, 30 min, F = 1.1, *P* = 0.36, 60 min, F = 2.6, *P* = 0.03, for 7th day were: at 15 min, F = 1.2, *P* = 0.30, 30 min, F = 5.1, *P* < 0.01, 60 min, F = 6.1, *P* < 0.01, whereas for 10th day were: at 15 min, F = 2.4, *P* = 0.05, 30 min, F = 1.9, *P* = 0.12, 60min, F = 2.2, *P* = 0.07, in all cases *df* = 5, 47. In the case where “-” is indicated, F and *P* values were not provided in the Table since no variances among the samples were recorded.
